# Risk stratification in non-ST elevation acute coronary syndromes: Risk scores, biomarkers and clinical judgment

**DOI:** 10.1016/j.ijcha.2015.06.009

**Published:** 2015-07-02

**Authors:** David Corcoran, Patrick Grant, Colin Berry

**Affiliations:** a West of Scotland Heart and Lung Centre, Golden Jubilee National Hospital, UK; b Department of Emergency Medicine, Glasgow Royal Infirmary, G4 0SF, UK; c BHF Glasgow Cardiovascular Research Centre, Institute of Cardiovascular and Medical Sciences, University of Glasgow, UK

**Keywords:** NSTE-ACS, Risk stratification, Myocardial infarction, GRACE score

## Abstract

Undifferentiated chest pain is one of the most common reasons for emergency department attendance and admission to hospitals. Non-ST elevation acute coronary syndrome (NSTE-ACS) is an important cause of chest pain, and accurate diagnosis and risk stratification in the emergency department must be a clinical priority. In the future, the incidence of NSTE-ACS will rise further as higher sensitivity troponin assays are implemented in clinical practice. In this article, we review contemporary approaches for the diagnosis and risk stratification of NSTE-ACS during emergency care. We consider the limitations of current practices and potential improvements.

Clinical guidelines recommend an early invasive strategy in higher risk NSTE-ACS. The Global Registry of Acute Coronary Events (GRACE) risk score is a validated risk stratification tool which has incremental prognostic value for risk stratification compared with clinical assessment or troponin testing alone. In emergency medicine, there has been a limited adoption of the GRACE score in some countries (e.g. United Kingdom), in part related to a delay in obtaining timely blood biochemistry results. Age makes an exponential contribution to the GRACE score, and on an individual patient basis, the risk of younger patients with a flow-limiting culprit coronary artery lesion may be underestimated. The future incorporation of novel cardiac biomarkers into this diagnostic pathway may allow for earlier treatment stratification. The cost-effectiveness of the new diagnostic pathways based on high-sensitivity troponin and copeptin must also be established. Finally, diagnostic tests and risk scores may optimize patient care but they cannot replace patient-focused good clinical judgment.

## 1 Introduction

Chest pain is a very common reason to attend the emergency department. Acute coronary syndrome (ACS) is a frequent cause of chest pain and ACS is associated with both a short, and a longer-term adverse prognosis.

The diagnosis of a non-ST elevation ACS (NSTE-ACS) in the emergency department should be followed by risk stratification and treatment. In this review article, we focus on clinical guideline recommendations for risk stratification and the challenges relating to the implementation of these guidelines in every day clinical practice. New developments and potential solutions are considered.

## 2 Background

Hospital attendances and admissions for acute chest pain present a substantial burden, accounting for 5% of all emergency department attendances, and 40% of acute medical admissions [1], [2]. Waiting times are a pressing societal problem and efficient treatment pathways are essential to ensure the accurate, timely and cost-effective early management of NSTE-ACS patients [3]. Conversely, missed diagnoses and treatment inefficiencies are associated with increased morbidity, mortality and costs [4].

ACS includes unstable angina (UA), non-ST elevation myocardial infarction (NSTEMI), and ST elevation myocardial infarction (STEMI) [5]. The diagnosis of ACS is based on the history of ischemic symptoms e.g. chest pain at rest, ischemic ECG changes, and elevated cardiac biomarkers, of which cardiac troponin (cTn) is the most commonly used. ACS has diverse causes, and the diagnosis of MI is classified according to the underlying cause [6] (Table 1).

**Table 1:** Classification of Myocardial Infarction.

Type 1
Spontaneous MI secondary to atherosclerotic plaque rupture
Type 2
MI secondary to ischaemic imbalance
Type 3
MI resulting in death when biomarker values are unavailable
Type 4a
MI related to percutaneous coronary intervention
Type 4b
MI related to stent thrombosis
Type 5
MI related to coronary artery bypass grafting

In contrast to evidence-based emergency provision of reperfusion therapy in STEMI [7], the treatment pathway for non-ST elevation ACS (NSTE-ACS) is more diverse. This heterogeneity includes variations in the levels of demand (e.g. absolute number of hospital admissions) and provision of treatment. Healthcare provision varies according to local resource availability (staff, beds, access to cardiac catheter laboratories), clinical guidelines (e.g. SIGN [8] and NICE [5] differ on definitions of clinical risk), and in their implementation (i.e. triage to cardiology or general medical wards). NSTE-ACS patients are also a diverse patient group, making them more challenging to diagnose and treat. Compared with STEMI, NSTE-ACS patients tend to be older and have more co-morbidity. Frailty and socio-economic problems are also important considerations [9].

## 3 Risk assessment of established or suspected NSTE-ACS

### 3.1 What the guidelines say

**Fig. 1:**
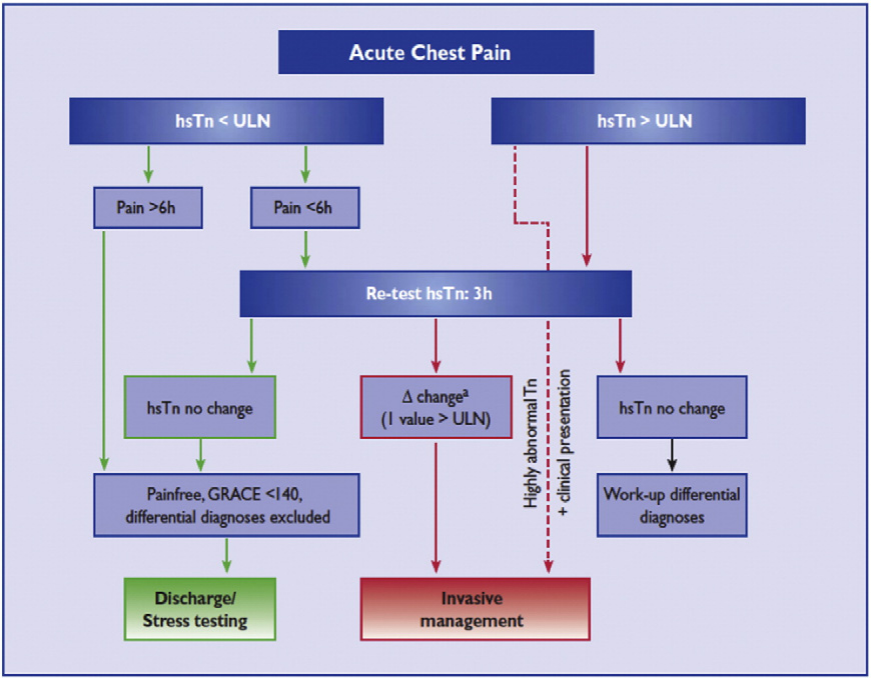
ESC rapid rule-out of ACS with hs-cTn algorithm.

International [10] and national [5] guidelines emphasize urgent invasive management of higher risk patients, such as defined by a Global Registry of Acute Coronary Events (GRACE) score for death or MI at 6 months greater than 140, on-going ischemia and/or hemodynamic instability, with early (< 24 h) or immediate invasive management [Fig. 1].

With permission of the Oxford University Press (UK)© European Society of Cardiology, www.escardio.org/guidelines.

### 3.2 Clinical diagnosis

Initial risk assessment is made clinically, based on the history, risk factors for coronary artery disease, clinical assessment (including the heart rate and blood pressure) [11], [12]. The ECG is recommended by the clinical guidelines as a first-line test that should be performed immediately. The ECG has good specificity (97%), but poor sensitivity (28%) in ACS [13]. This assessment is of pivotal importance since cardiac biomarkers are usually unavailable initially.

### 3.3 Biomarkers

Since their introduction over 20 years ago, cTn assays have become the established investigation for the detection of myocardial necrosis in the work-up of patients with suspected NSTE-ACS [14]. The assays used have become increasingly more sensitive for the diagnosis of MI [15]. Despite this, biomarkers other than cTn have been investigated. The ideal cardiac biomarker would be present only in myocytes, be released early after necrosis, have a value proportional to the myocardial infarct size, and be detectable for a number of days with a predictable clearance [16], [17]. It should be practical for use in the emergency department and cost-effective [18].

The emergence of high-sensitivity troponin (hs-cTn) assays is an important advance for diagnostic testing for ACS. Other potential biomarkers include heart-type fatty acid-binding protein (H-FABP); biomarkers of systemic inflammation such as CRP; markers of LV dysfunction, namely brain natriuretic peptide (BNP); and copeptin and ischemia-modified albumin [19].

A new biomarker in NSTE-ACS should have incremental diagnostic and prognostic value, and be cost-effective. Their role may be to improve upon the poor early sensitivity of cTn and in the further risk stratification of patients with a negative second cTn. The combination of cTn and copeptin could potentially reduce inappropriate admission to hospitals [20], and novel biomarkers may improve risk stratification of cTn-negative patients [21]. So far, testing of novel biomarkers at presentation increases sensitivity at the expense of reduced specificity. Still, a multi-biomarker approach might not obviate the need for stress testing on clinical grounds to help clinicians explain the origin of a patient's symptoms [22]. The combination of hs-cTn and novel biomarkers has also been investigated. In a prospective observational study of 478 patients, the combination of hs-cTn and copeptin demonstrated significantly higher sensitivity to identify NSTE-ACS than a repeat hs-cTn [23].

### 3.4 High-sensitivity cardiac troponin

hs-cTn assays are defined as having an imprecision at the 99th centile ≤ 10%, and cTn concentrations below the 99th centile can be detected in ≥ 95% of normal individuals. Troponin levels as low as 3 ng/L may now be detected compared to 0.05 μg/L for the third-generation assays [24]. A clinical strategy based on hs-cTn testing in the emergency department will increase the detection rate for NSTEMI, and potentially enable the earlier use of evidence-based therapies to prevent ischemic complications. Such a strategy might also facilitate earlier stress testing or discharge of patients from the emergency department [25], [26].

Based on increased sensitivity, hospital admission for a 12h cTn should generally not be required, but a second test might be reliable at 6h leading to a sensitivity of 99–100% [6]. The optimal timing of a second test will most likely be related to the initial likelihood of coronary disease based on clinical characteristics, and future population-level studies will be needed to work this out. Serial sampling and a rising hs-cTn concentration, or a ‘delta value’ of 20%, may improve specificity [10], and hs-cTn may add incremental value to the discrimination of the GRACE score [27]. The current European Society of Cardiology (ESC) consensus guideline on the use of hs-cTn recommends that for patients with a negative initial hs-cTn or values close to the upper reference limit URL, a 50% delta value or an absolute increase of 7 ng/L at 3 h can be used to diagnose MI. In patients with elevated hs-cTn at presentation, a delta value of 20% at 3 h can be used [28]. Overall, the increase in sensitivity of the diagnostic tests will inevitably contribute to increases in the detection and incidence of NSTEMI [29], [30].

Unfortunately, with the increased sensitivity there is a reduction in specificity, with a reported value of 80–85% leading to higher false-positive values [31]. hs-cTn is specific for myocardial injury but not ACS. Low-level detectable troponin may be present due to chronic kidney disease, supposed stable coronary artery disease or even exercise-induced transient ischemia. cTn release may also be secondary to other conditions such as pulmonary thrombo-embolism or myocardial oxygen-perfusion mismatch leading to a Type 2 MI (e.g. new onset atrial fibrillation with an uncontrolled ventricular rate) [32]. Such increases in hs-cTn should not be interpreted as clinically insignificant. In a recent cohort study of consecutive patients admitted to an acute medical unit over one month, 564 patients had hs-cTn measured on admission, representing approximately 50% of all admissions. Of those who had hs-cTn measured, 40% had a result > 14 ng/l, but only 20% had a final diagnosis of MI. In spite of this, mortality was 31% at one year, with a tendency towards worse outcome in those with a final diagnosis other than MI [33].

### 3.5 Novel biomarkers

#### 3.5.1 Copeptin

Copeptin is the C-terminal part of the arginine vasopressin (AVP) precursor. Copeptin is a surrogate marker for AVP, which is elevated in NSTE-ACS, but whose utility is limited by a short half-life. It is postulated that AVP is released as part of the endocrine stress axis response to ACS. Copeptin levels rise rapidly at 0–4h and decline over 2–5 days. A copeptin assay was subject to a NICE medical technology guidance report which recommended that further clinical studies were required to assess its utility. In the largest trial to date in 2013, copeptin testing facilitated the exclusion of NSTEMI within 4h of attendance at the emergency department [34]. The combination of a negative copeptin and a negative troponin increased the negative predictive value to 99% [35], which may help to reduce the time taken to rule out myocardial infarction if introduced into clinical practice.

### 3.6 H-FABP

H-FABP is an intracellular protein involved in myocardial fatty acid metabolism, but is also expressed in lower levels in other tissues [36]. It is rapidly detectable following myocardial ischemia and infarction, but has not been shown to have incremental diagnostic value in the diagnosis of MI when combined with hs-cTn [37].

### 3.7 Cost-effectiveness

The cost-effectiveness of new biomarkers in the ACS diagnostic pathway is paramount, as highlighted by the Health Technology Assessment for the National Institute for Health Research (NIHR) [21]. As would be expected, admission for a second cTn level was not found to be cost-effective unless it could be acted upon rapidly when compared with hs-cTn testing on presentation and a second level at 3h. The latter strategy could facilitate the early discharge of patients from the emergency department prior to the 4h target.

Regarding novel biomarker testing at presentation, the addition of H-FABP, copeptin and myoglobin demonstrated increased sensitivity at an acceptable QALY threshold, but more evidence is needed.

### 3.8 Risk scores

#### 3.8.1 Current approaches and challenges with initial risk stratification

Risk estimation by physicians on clinical grounds alone correlates poorly with observed outcomes [38]. Despite being associated with poorer prognosis, troponin per se is not an accurate measure of risk when compared to risk scores which incorporate clinical variables, the ECG, and biomarkers [39].

Once a diagnosis of NSTE-ACS is suspected and/or confirmed, clinical guidelines recommend risk stratification with the GRACE score [5], [10]. It was derived from a large registry of ACS patients (n = 11,389) to predict death and death or MI in-hospital and at six months [40], [41], [42]. The web-based calculator (or ‘app’) is simple to use and the result is expressed as a probability (%) value or a score.

In order to complete the GRACE score, blood chemistry results are needed, which take time to obtain during clinical care, especially if paired tests are performed, separated by a 12h interval. This delay means that NSTE-ACS patients are normally admitted to the hospital, at least temporarily.

### 3.9 GRACE score

GRACE 1.0 score has been extensively validated [10]. GRACE 1.0 predicts the in-hospital and 6-month risk of death or MI using the variables: age, heart rate, systolic blood pressure, creatinine, Killip classification of heart failure, ST-segment deviation, elevated cTn, and the occurrence of cardiac arrest on admission. The ESC and NICE guidelines recommend using this score to identify those patients who will benefit from an invasive strategy. The recently implemented GRACE 2.0 score implements revised GRACE algorithms for predicting death and death or MI at 1 and 3 years, and is now defined as a medical device (available at http://www.gracescore.org/WebSite/WebVersion.aspx).

### 3.10 Limitations of the GRACE score

#### 3.10.1 Partial adoption

The GRACE risk score is only partially used in real-life clinical practice [43]. In an audit conducted in our hospital in Glasgow over a four-month period in 2011, only 34.5% of all patients (n = 55) referred from the emergency department with a diagnosis of ACS had a GRACE score calculated and recorded in the case notes (unpublished data). Despite efforts to improve adoption e.g. with an acute chest pain proforma, further audit indicates that adoption has not changed. Peer feedback from cardiologists in other European hospitals also indicates partial adoption. Suboptimal risk stratification in the emergency department may lead to non-evidence-based treatment decisions and transfer to inappropriate wards.

The reasons for non-adoption of the GRACE score in daily practice include a lack of awareness by medical and nursing staff, a lack of access to the on-line website, and lack of time in a busy department since the creatinine and troponin blood results are required before the score can be completed. One other limitation could be that the GRACE tool has different numeric outputs, as a percentage risk and a score, which may lead to user uncertainty. A recent qualitative study of health practitioners at 11 hospitals throughout The Netherlands on the use of risk scores highlighted similar issues [44].

Some solutions to these problems include education of medical and nursing staff in the emergency department, the GRACE score ‘app’ for handheld devices, bookmarked access to the GRACE website, and also hospital admission documents which require the GRACE score to enable appropriate in-patient stratification.

The Myocardial Ischaemia National Audit Project (MINAP) registry of patients admitted in England and Wales with ACS collects six of the eight variables of the GRACE score. To assess the performance of the GRACE score in the MINAP registry, NICE produced a ‘mini-GRACE’ score to allow the calculation of a GRACE risk score without the missing variables (a creatinine value or Killip class). This score was subsequently modified to produce the ‘adjusted’ mini-GRACE score using the MINAP variables ‘creatinine above 200 or not’, and ‘loop diuretics prescribed during admission’ as surrogates for creatinine and Killip class [45]. This is available online as the ‘GRACE 2.0 Risk Calculator’, and allows risk stratification without a creatinine, but there is still a need to wait for the cTn value. This score correlates well in the NICE analysis, but performed less well in the highest risk patients.

#### 3.10.2 Limitations with accuracy

Age has an exponential contribution to the GRACE score. For example, a young cigarette smoker (e.g. 35 year old male) without ischemic ECG changes or other abnormalities but with an elevated troponin concentration may still have a relatively low GRACE score (e.g. 87 for death/MI at 6 months). Alternatively, an elderly person with a troponin elevation and non-specific fixed ECG changes will have a GRACE score greater than 140. According to clinical guidelines from NICE, the younger patient would be managed medically whereas the older patient could be referred for urgent invasive angiography. Clearly, clinical judgment is important [46]. Finally, chest pain patients with suspected but unconfirmed ACS may be unnecessarily exposed to the risks of anti-thrombotic drug therapies, especially if the final diagnosis is a non-ischemic etiology.

### 3.11 TIMI and PURSUIT scores

The TIMI [47] and PURSUIT [48] risk scores have also been widely validated, but GRACE has superior discriminative value for prognostication [5].

### 3.12 Treatment of suspected rather than confirmed NSTE-ACS

Clinical guidelines recommend prescription of evidence-based anti-thrombotic therapies including aspirin, a P2Y_12_ inhibitor, and anti-coagulation when an ACS appears likely [10]. Conversely, there is a bleeding risk associated with these drugs, meaning the initial risk–benefit ratio in low risk NSTE-ACS patients is less clear. Bleeding complications in NSTE-ACS are associated with a poorer prognosis and bleeding scores have been developed to help inform the likelihood of bleeding [10]. However, bleeding risk scores are infrequently used in daily practice.

Audit data in our hospital indicates that dual anti-platelet therapy including ticagrelor has been administered at the time of the first medical contact in patients with suspected but unconfirmed NSTE-ACS, and in some of these patients the final diagnosis was non-cardiac. Since an expectant approach to prescription of triple anti-thrombotic therapy could expose patients to unnecessary bleeding complications (especially in the elderly) and incur sub-optimal use of secondary care resources (i.e. morbidity related to bleeding complications), our practice now restricts P2Y_12_ inhibitor therapy to patients with a confirmed ACS.

### 3.13 Bleeding scores

Major bleeding in ACS is associated with worse outcome [49]. Bleeding scores have a class 1B recommendation in the ESC guidelines, however they are rarely used in clinical practice. The intention is that these scores will predict the baseline bleeding risk, which can be interpreted together with a risk stratification score such as GRACE, in order to define a patient's treatment strategy and the anti-thrombotic therapies prescribed.

The most frequently used is the CRUSADE bleeding score which assigns patient a number of points from 1 to 100 based on 8 independent predictors of major in-hospital bleeding [50]. This score was subsequently validated in 17,857 patients admitted with NSTE-ACS [51]. An online calculator is available at http://www.crusadebleedingscore.org. With the introduction of the newer P2Y_12_ inhibitors, the increasing use of the novel oral anti-coagulants, and the development of agents such as the PAR-1 agonists, bleeding risk assessment will continue to evolve.

### 3.14 Problems with diagnostic tests

In order to facilitate the clinically-appropriate rapid discharge from the emergency department of patients who present with low-intermediate risk chest pain, and conversely to triage appropriate NSTE-ACS patients to Cardiology beds, stress and non-invasive imaging modalities have been evaluated in the acute setting. A significant regional variation in their use exists depending on local expertise and availability.

### 3.15 Exercise treadmill testing

Clinical guidelines do not recommend routine exercise treadmill testing (ETT) for risk stratification of NSTE-ACS patients. However, clinical practice often differs from guidelines, and treadmill exercise testing is still widely used in some countries, including the United Kingdom. This is especially true in hospitals with high admission rates, limited bed capacity, and where demand for invasive angiography exceeds capacity. Clinicians may use the treadmill test to facilitate the discharge of low-intermediate risk medically stabilized NSTE-ACS patients [8].

### 3.16 CT coronary angiography

CTCA has been proposed as a modality to improve the early management of NSTE-ACS patients. In the largest study to date, involving 1000 patients at 9 hospitals in the United States, CTCA facilitated the exclusion of a diagnosis of ACS, increasing discharge from the emergency department and reducing length of stay [52]. This study was performed during normal working hours, and widespread adoption of CTCA could be limited by lack of availability out-with office hours. Alternatively, a diagnostic pathway based on CTCA might in fact necessitate potentially unnecessary admissions to hospital if out-of-hours imaging is not available. CTCA involves exposure to ionizing radiation which may be minimized by dose-limiting techniques such as prospective gating. However, in reality, high heart rate and rhythm variations often preclude prospective gating or other measures to reduce radiation exposure (e.g. padding) such that a retrospective approach with higher radiation doses is commonly used. In fact, retrospective CTCA involves radiation exposure that is at least as high as invasive angiography. Finally, although CTCA has high negative predictive value, the positive predictive value is inferior to invasive angiography. CTCA is not evidence-based for risk stratification of NSTE-ACS. However, its non-evidence based use for this purpose in every-day practice in some hospitals (especially hospitals without invasive angiography) should be a matter of some concern.

For the heterogeneous population of troponin-negative patients, there is some evidence that non-invasive CTCA coronary angiography (CTCA) in a selected population with a higher underlying risk of adverse events may be cost-effective. One proposed strategy is the stratification of troponin-negative patients with novel biomarker testing, with those at higher risk undergoing CTCA [21].

### 3.17 Stress imaging

In contrast to anatomical non-invasive imaging, functional stress imaging has also been investigated to aid diagnosis and risk stratify patients presenting acutely with chest pain suspicious of ACS, but who are troponin-negative and do not have diagnostic ECG findings.

Stress echocardiography has previously been shown to be superior to ETT in the risk stratification of patients presenting acutely with chest pain [53]. In the largest study to date, 839 consecutive patients who had a non-diagnostic ECG and negative 12h cTn were risk stratified with use of dobutamine stress echocardiography. Rapid discharge and accurate risk stratification were demonstrated for this modality in a chest pain unit setting [54]. As with CTCA, this study was performed within working hours, meaning that patients admitted over the weekend had a longer length of stay.

Single photon emission computed tomography (SPECT) has also been validated in the acute chest pain setting using rest-only and stress-only protocols [55], [56]. However, as with CTCA, ionizing radiation is required, and availability is limited for the high throughput of patients required to facilitate discharge from the ED. Similarly, stress perfusion cardiac MRI is limited by availability and high initial cost in this setting, but has been demonstrated to be feasible and effective at reducing admissions [57].

### 3.18 Stratification to invasive management

**Fig. 2:**
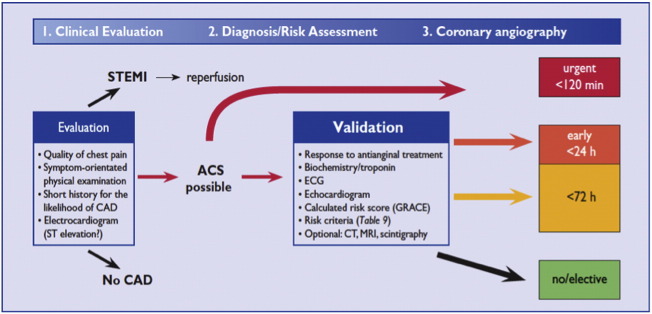
ESC decision-making algorithm in ACS.

Clinical guidelines recommend risk stratification of NSTE-ACS patients with the GRACE score once the diagnosis has been established. The ESC guidelines recommend that patients with a GRACE of greater than 140 should undergo coronary angiography within 24h [10], and for very high risk patients the angiogram should be performed within 2h. Patients with NSTE-ACS who are assessed as lower risk (e.g. GRACE score < 140) invasive management should be performed within 72h. [Fig. 2].

With permission of the Oxford University Press (UK)© European Society of Cardiology, www.escardio.org/guidelines.

NICE [5] recommends that patients with an intermediate risk (GRACE scores 88–100, representing a 6-month mortality > 3.0%) or higher should be triaged for an invasive strategy, with coronary angiography performed within 96h with follow-on PCI or CABG as appropriate. Medically stabilized lower risk patients (e.g. GRACE score < 88 as per the NICE guidelines) may be discharged with planned outpatient angiography, as clinically appropriate [5].

A meta-analysis of three large trials investigating a routine invasive versus selective invasive strategy in patients with NSTE-ACS namely FRISC, RITA-3, and ICTUS, which themselves had shown conflicting results, demonstrated benefit of a routine invasive strategy, driven predominantly by a reduction in MI. The largest benefit was seen in those at highest risk, defined by the GRACE score [58]. The TIMACS trial, which compared an early (within 24h), with a delayed (minimum of 36h) invasive strategy did not show a benefit of routine early coronary angiography within 24h, except within the subgroup of patients with a GRACE risk score at admission for death or MI at 6 months of greater than 140, who were deemed high risk [59]. This trial result supports the stratification of treatment based on the GRACE score, although the evidence-base is limited. It should be remembered however that the GRACE score was developed to predict in-hospital and 6-month mortality, and not the utility of undergoing invasive management.

### 3.19 Delays to coronary angiography

**Fig. 3:**
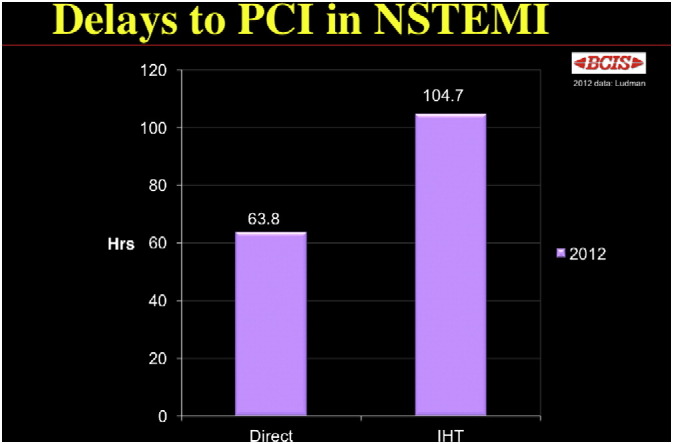
Delay to PCI in those patients admittedly directly to an interventional hospital compared with those requiring inter-hospital transfer (IHT). BCIS Audit Returns 2012.

In practice, achieving invasive management within the recommended timelines is difficult, as indicated by the British Cardiovascular Intervention Society National Audit (2012) [60]. Overall, 65.9% of patients referred for early invasive management had coronary angiography performed within 96h. For patients initially admitted to a non-interventional hospital and who require inter-hospital transfer to a tertiary centre for coronary angiography, the average time to angiography was 104.7h, which is nearly double the time-to-angiography for patients admitted to a hospital with interventional cardiology on site (63.8h) [Fig. 3].

Reprint permission granted from Dr. Peter Ludman, Consultant Cardiologist, Queen Elizabeth Hospital, Edgbaston, Birmingham B15 2TH, UK; peter.ludman@uhb.nhs.uk.

In contrast to the widespread 24/7 availability of primary PCI for STEMI patients, provision of invasive management for higher risk NSTEMI patients varies widely. Some European hospitals transfer NSTEMI urgently to the catheter laboratory in just a few hours in line with clinical guidelines whereas many hospitals e.g. in the United Kingdom, provide a routine office hour service. As such, a patient who is admitted on a Thursday evening or Friday and has medically-stabilized symptoms may wait until the following week for coronary angiography. A retrospective study of 1190 patients admitted with ACS in Edinburgh demonstrated that patients waited longer for in-patient coronary angiography (2.7 vs. 2.0 days) if they were admitted to a district general hospital with no on-site cardiac catheterization laboratory compared with admission to an interventional center [61]. A similar delay was demonstrated in an audit undertaken in New Zealand in 2010, with a longer median wait (5.1 vs. 2.5 days) for patients admitted to a non-interventional hospital [62].

## 4 Conclusion

The clinical and health economic utility of non-invasive imaging in a population with suspected ischemic chest pain in routine hospital practice has been called into question [63] and the value of physician discretion in decision making has again been emphasized [64]. There can be no substitute for good clinical judgment, based on the acquired diagnostic skills, knowledge of the literature, and clinical experience. Risk scores and tests may complement but not replace patient-focused treatment decisions by the emergency medicine staff.

The optimal risk stratification of NSTE-ACS patients is a key priority in emergency medicine. Risk stratification based on prognostic scoring, such as GRACE, improves the selection of higher-risk patients for invasive management. However, the accuracy of diagnostic strategies is key to preventing inappropriate hospital admissions, minimizing avoidable morbidity and costs. The role for novel biomarkers in addition to cTn has yet to be established, but may help to define those who are truly low risk and who can be safely discharged from the ED. Given their limitations, these tools will assist but not replace clinical judgment.

## Conflict of interest

Professor Berry has received reimbursement for lectures on anti-thrombotic therapies supported by AstraZeneca and Bristol-Myers Squibb and was supported by a Senior Clinical Fellowship from the Scottish Funding Council.
